# Sleep Improvement for Metabolic Health: A Feasibility Trial of a Digital Sleep Treatment in People With Insomnia and Non‐Diabetic Hyperglycaemia

**DOI:** 10.1111/jsr.70306

**Published:** 2026-02-15

**Authors:** Rachel Sharman, David Ray, Andrew Farmer, Poppy C. E. Green, Victoria Harris, Fredrik Karpe, Colin A. Espie, Diana Mantripp, Thomas Marjot, Niall M. McGowan, Jeremy W. Tomlinson, Simon D. Kyle

**Affiliations:** ^1^ Sir Jules Thorn Sleep and Circadian Neuroscience Institute Nuffield Department of Clinical Neurosciences, University of Oxford Oxford UK; ^2^ Oxford Centre for Diabetes, Endocrinology and Metabolism University of Oxford Oxford UK; ^3^ Nuffield Department of Primary Care Health Sciences University of Oxford Oxford UK; ^4^ NIHR Oxford Biomedical Research Centre, OUH Trust Oxford UK

**Keywords:** digital cognitive behavioural therapy, insomnia, metabolic dysfunction, non‐diabetic hyperglycaemia, pre‐diabetes

## Abstract

Insomnia may play a causal role in type 2 diabetes (T2D). Addressing insomnia through cognitive behavioural therapy (CBTi) in people with non‐diabetic hyperglycaemia could potentially reduce the risk of progression to T2D. To inform a future randomised trial, we performed a feasibility study of digital CBT (dCBTi) in individuals at increased risk of T2D. Participants were identified from 10 primary care practices in the UK and given access to dCBTi. Outcomes were evaluated at baseline (Week‐0) and post‐treatment (Week‐11). Primary feasibility outcomes were ability to recruit and treatment engagement. We also quantified within‐group mean change (95% CI) in insomnia severity (Insomnia Severity Index), health‐related quality of life (EQ‐5D‐3L), depression (Center for Epidemiologic Studies Depression Scale), chronotype (reduced Morningness‐Eveningness Questionnaire), sleep (7‐day actigraphy and diary), continuous glucose monitoring (7‐days) and fasting blood metabolites (insulin, lipids, glucose and C‐reactive protein). The recruitment target was 20. Of 242 people completing screening, 36 were eligible and 24 were enrolled (age 65.5 ± 12.4 years, 70.8% female). Twenty‐three (96%) completed post‐intervention assessments. Treatment engagement was excellent (83.3% completed ≥ 4 sessions). The intervention was associated with a large reduction in insomnia severity [−4.7 (95% CI: −6.2 to −3.2), *d* = −1.4] and medium reduction in depressive symptoms [−2.7 (95% CI: −5.1 to −0.2), *d* = −0.5]. Sleep diary parameters tended to show greater improvement following intervention relative to actigraphy. There was evidence of a reduction in serum lactate, glycerol and triglycerides but no clear change in glucose or insulin. Results suggest a full trial is likely feasible and that people with NDH find the intervention acceptable and beneficial.

**Trial Registration:** This trial was prospectively registered on the UKs clinical study registry, the ISRCTN (ISRCTN19682964, https://doi.org/10.1186/ISRCTN19682964)

## 1 Introduction

Diabetes mellitus is a highly prevalent disorder and a leading cause of morbidity and mortality (Lin et al. 2020). In 2022 an estimated 828 million individuals worldwide were living with diabetes, with type 2 diabetes (T2D) accounting for over 80% of cases (Ong et al. 2023; Zhou et al. 2024). This figure is expected to increase to 1.3 billion by 2050, with rates rising higher in children and those under 40 years of age (Ahmad et al. 2022; Lascar et al. 2018; Ong et al. 2023). T2D is associated with a myriad of long‐term health conditions, including vascular disease, heart disease, kidney disease, retinopathy and neuropathy (Ahmad et al. 2022). Within the UK, T2D poses a substantial financial burden on the National Health Service (NHS), consuming around 10% of the annual healthcare budget (Whicher et al. 2020).

Sleep plays a key role in cardiometabolic function. Prospective data indicates that sleep duration (both short and long) is associated with a greater risk of diabetes (Cappuccio et al. 2010; Chao et al. 2011; Shan et al. 2015). Further, experiencing insufficient sleep due to later sleep onset times and an evening chronotype is associated with an increased risk of T2D (Knutson and von Schantz 2018; Ma et al. 2022; Shen et al. 2025). In non‐obese, healthy participants, experimental sleep restriction and sleep fragmentation drive change in factors associated with diabetic status, including increased hunger, reduced insulin sensitivity/increased insulin resistance, increased postprandial glucose surges and increased cortisol (Reynolds et al. 2012; Stamatakis and Punjabi 2010; Sweeney et al. 2017; Zhu et al. 2019; Zuraikat et al. 2024). Conversely, experimental sleep extension in short sleepers improves metabolic parameters and weight reduction (Hartescu et al. 2022; Leproult et al. 2015; So‐Ngern et al. 2019; Tasali et al. 2022).

Insomnia is a prominent comorbidity in those with T2D, affecting 39% of patients (Schipper et al. 2021; Koopman et al. 2020). Conversely, insomnia increases risk for developing T2D (Wright et al. 2025). This risk is heightened in younger participants (under 40 years of age); (Lin et al. 2018). Mendelian randomisation also shows that genetic instruments for frequent insomnia symptoms are causally related to elevated glycated haemoglobin (HbA_1C_) (Liu et al. 2022; Ma et al. 2022; Yuan and Larsson 2020) and that insulin resistance is a significant mediator of the relationship between insomnia and T2D (Wang et al. 2023).

If insomnia is causally related to hyperglycaemia, then effective intervention could improve metabolic health. A recent meta‐analysis found three studies that have assessed the effects of CBTi on glycaemic measures in people with insomnia, finding some evidence of improvement in HbA_1C_, albeit with small effect sizes (Mostafa et al. 2025). More broadly, several reviews have shown that non‐pharmacological sleep interventions can generally improve glycaemic outcomes, but small and heterogeneous samples and poor treatment engagement were identified as factors influencing the quality and robustness of evidence to date (Mostafa et al. 2025; Savin et al. 2023).

No study has assessed the effects of sleep intervention in non‐diabetic hyperglycaemia (NDH), a key risk state for diabetes characterised by raised (though sub‐clinical) glucose concentrations. NDH represents a distinct risk state for type 2 diabetes, with specific behavioural and clinical characteristics that may affect recruitment, engagement and intervention uptake. Studies have successfully recruited participants with NDH from GP practice records in the UK (e.g., Davies et al. 2016) but, to our knowledge, no study has explored recruitment of individuals experiencing both NDH and sleep difficulties to take part in a sleep improvement programme.

To address this gap, we conducted a feasibility trial of digital cognitive behavioural therapy (dCBT) in individuals with insomnia and NDH to inform a future large‐scale RCT. The primary aim of the study was to evaluate the feasibility of recruiting eligible participants with both insomnia and NDH from primary care to the trial. Secondary outcomes were to explore adherence to the intervention protocol and provide proof‐of‐principle data regarding changes in sleep and metabolic outcomes.

## 2 Materials and Methods

### 2.1 Participants

Ten primary care practices within Oxfordshire, UK, were identified and approached to identify participants. Practice medical records were searched using a preset screening algorithm to identify potential participants. Potentially eligible participants were identified as being aged 18 years or over, having either an HbA1c of 6%–6.4% within the past 12 months or a diagnosis of NDH (pre‐diabetes), no current diagnosis of diabetes or current use of diabetic medication, no recorded diagnosis of sleep apnoea and no current hypnotic prescription.

All patients within the practice who met these initial search criteria were sent a letter and participant information sheet and invited to complete an online screening questionnaire (Qualtrics, Provo, UT) to further assess eligibility. Inclusion criteria included meeting DSM‐5 criteria for insomnia disorder according to the Sleep Condition Indicator (SCI (Espie et al. 2014)), having reliable internet access, and being able to understand the study instructions in English. Participants were excluded if they had evidence of another sleep disorder, including obstructive sleep apnea (OSA, assessed with the STOP‐BANG (Chung et al. 2016)), narcolepsy, restless legs syndrome (RLS)/periodic limb movement disorder, circadian rhythm sleep–wake disorder or parasomnia (Espie 2024; Wilson et al. 2010); psychosis or epilepsy; prescribed diabetes or sleep medication; engaged in shift work; dementia or mild cognitive impairment; suicidal ideation with intent; pregnant or planning pregnancy; active cancer; planned surgery in the next 2 months; life expectancy of less than 1 year; previous or current access to the digital CBTi programme (Sleepio); currently receiving psychological therapy for insomnia or engaged in another sleep intervention trial; or had an allergy to hypoallergenic adhesive plasters.

The study was approved by the Health Research Authority (IRAS 266313) and the allocated Research Ethics Committee (East of England—Essex Research Ethics Committee, 20/EE/0046) and was prospectively registered on the ISRCTN (ISRCTN19682964).

### 2.2 Procedures

All study procedures took place at the NIHR Oxford University Hospitals Biomedical Research Centre Clinical Research Unit, within the Oxford Centre for Diabetes, Endocrinology and Metabolism, UK (OCDEM CRU). Once eligibility was confirmed, participants were invited to a baseline research appointment with a research nurse where they gave informed consent to the trial, had a blood sample drawn, completed a cognitive task and questionnaires, were fitted with a continuous glucose monitor (CGM: Dexcom, California, USA), were given a sleep diary (Consensus Sleep Diary: (Carney et al. 2012)), and a wrist‐worn actigraphy device (MotionWatch8, CamNtech, UK). A research nurse completed CGM placement to ensure standardisation between participants, affixing the device to the participant's abdomen.

For the next 7 days, participants wore the CGM and actiwatch and completed the sleep diary. They then returned to the research unit to have the CGM removed and return the actiwatch and sleep diary. Immediately following this, participants were given access to the sleep intervention to complete over 10 weeks. At Week 11, participants returned to OCDEM CRU for the post‐assessment visit, which followed the same procedures as the baseline visit. Participants were reimbursed with a £50 voucher for participation in the trial, alongside travel expenses. For the study flow, see Figure 1.

**FIGURE 1:**
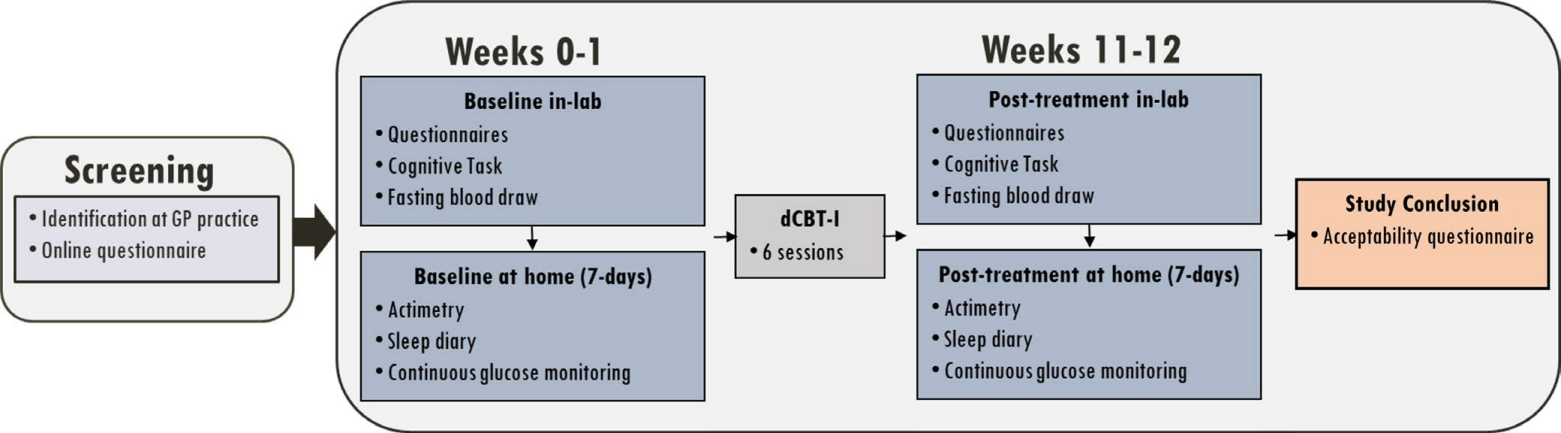
Study flow diagram demonstrating the flow of procedures and measurements for the SleepTMH study.

### 2.3 Intervention

The digital cognitive behavioural therapy for insomnia (dCBTIi) intervention was delivered using the Sleepio programme, a NICE‐approved and FDA‐cleared fully automated programme, shown to be clinically effective (Espie et al. 2012). The six‐session programme was completed independently over a 10‐week period. The treatment includes a behavioural component (sleep restriction, stimulus control and relaxation), a cognitive component (paradoxical intention, cognitive restructuring, mindfulness, positive imagery and putting the day to rest) and an educational component (psycho‐education and sleep hygiene). Each session lasts for around 20 min and the content of the programme is presented by an animated virtual therapist. The programme includes a daily sleep diary which is used by the programme to create personalised help and prescribed sleep–wake schedules. The system provided online analytics, which can be used to monitor engagement by assessing how many sessions were completed and the number of weeks to complete the course.

### 2.4 Outcome Measures

#### 2.4.1 Primary Outcome—Feasibility

The primary outcome was the feasibility of recruiting eligible participants from a primary care setting. For this, recruitment rates per month were calculated.

#### 2.4.2 Secondary Outcomes

##### 2.4.2.1 Acceptability

Acceptability was indexed by the proportion of participants who completed at least four of the six intervention sessions within the 10‐week period.

##### 2.4.2.2 Treatment Satisfaction

At the Week 11 visit, participants completed a treatment satisfaction questionnaire consisting of two questions assessed on a 5‐point Likert scale. Participants were asked ‘Overall, how satisfied are you with the sleep treatment you received?’ scored from 0 ‘Very Dissatisfied’ to 4 ‘Very satisfied’. Perceived sleep improvement was captured by the item ‘To what extent did the sleep treatment improve your sleep?’ scored from 0 ‘Not at all’ to 4 ‘A lot’.

##### 2.4.2.3 Proof of Principle Outcome Measures

Proof‐of‐principle outcome data were collected to inform a full RCT. Measures were collected at Weeks 0–1 (baseline) and Weeks 11–12 (post‐assessments).

Insomnia severity was measured by the Insomnia Severity Index (ISI: (Morin 1993)), depressive symptomology was measured by the Centre for Epidemiologic Studies Depression Scale (CES‐D: (Radloff 1977)), chronotype was measured by the Morningness‐Eveningness Questionnaire (reduced version, rMEQ: (Adas and Alwrall 1991)), health‐related quality of life was measured by EuroQol (EQ‐5D‐3L: (Kind et al. 1998; The EuroQoL Group 1990)) and overall self‐rated health was assessed by the visual analogue scale from the EQ‐5D (EQ‐5D‐3L VAS).

Subjective sleep was assessed for 7 days at pre‐ and post‐treatment using the Consensus Sleep Diary (CSD: (Carney et al. 2012)). Outcomes were: time in bed (TIB); total sleep time (TST); wake after sleep onset (WASO); sleep efficiency (SE); sleep onset latency (SOL); and sleep quality (scored on a 5‐point Likert scale from 0 ‘very poor’ to 4 ‘very good’). We also assessed cognitive arousal each morning using item five (‘last night, as you were attempting to fall asleep or return to sleep was your mind mentally alert and active’) from the pre‐sleep arousal scale (PSAS: (Nicassio et al. 1985)) scored on a 5‐point Likert scale from 0 ‘not at all’ to 4 ‘extremely’.

Estimated objective sleep parameters and rest‐activity rhythms were derived from actigraphy (MotionWatch 8, CamNtech) recorded over a seven‐day collection period at 30‐s epochs on a medium sensitivity. Outcomes were: TIB; TST; WASO; SE; SOL; relative amplitude of the rest activity cycle (RA); interdaily stability of the rest activity cycle (IS); and intradaily variability of the rest activity cycle (IV).

A fasted blood sample was collected from participants at Weeks 0 and 11 assessments. Participants were instructed to fast from 10 PM the night before their visit. Each blood sample was 9.5 mL of whole blood. Blood samples were analysed for HbA_1c_, insulin, glucose, non‐esterified fatty acids (NEFA), glycerol, lactate, urea, triglycerides, low‐density lipoprotein cholesterol, high‐density lipoprotein cholesterol, beta‐hydroxybutyrate, apolipoprotein‐B, alanine transaminase and C‐reactive protein.

Free‐living interstitial glucose concentrations were recorded with a continuous glucose monitoring system (CGM; Dexcom G6). The CGM was affixed to the participant's abdomen and measured interstitial glucose levels every 5 min over a 1‐week recording period, concurrent with the measurements above. The CGM device was utilised in a blinded mode, meaning participants did not receive live feedback on their glucose levels. However, for safety, alarms were set to sound if blood sugar levels reached an extreme ‘high’ or ‘low’ during the recording period.

Working memory performance was assessed at Weeks 0 and 11 using the visuospatial working memory task (VSTM) (Zhao et al. 2025). The VSTM takes around 10 min to complete. Participants are presented with 1 or 3 fractal patterns on the screen. After a 4‐s delay, one of the previously presented fractal patterns are displayed alongside a foil fractal (one not presented previously). Participants are required to identify the true presented fractal pattern and then move the fractal to the position where it originally appeared on the screen. This task probes both identification memory and localisation memory (number correctly identified fractals, identification reaction time and localisation reaction time).

##### 2.4.2.4 Adverse Events

Participants were asked to report on accidents and injuries occurring within the past 2 months, both pre‐ and post‐intervention. This 5‐item questionnaire included 3 binary (yes/no) items, seeking responses to whether the participant had experienced a work‐related accident, a motor vehicle accident or a near‐miss driving incident within the past 2 months. The final two items asked the participant to report how many times they had fallen asleep while driving and how many times they had experienced a fall within the past 2 months.

During study visits, nurses enquired about any serious adverse events, defined as any untoward medical occurrence that results in death, is life‐threatening, requires inpatient hospitalisation or prolongation of existing hospitalisation, results in persistent or significant disability/incapacity or consists of a congenital anomaly or birth defect.

## 3 Statistical Analysis

A statistical analysis plan was drafted and finalised prior to final data lock. Data were analysed by a statistician (VH) using STATA (StataCorp. 2025. Stata Statistical Software: Release 19. College Station, TX: StataCorp LLC). Initial processing of actigraphy data was conducted in the proprietary MotionWare software. CGM data was processed using the iglu R package (Broll et al. 2021) and consistent with the recommendations of the Lancet Diabetes & Endocrinology consensus statement (Battelino et al. 2023).

The primary outcome measure included all participants who consented to the trial. Participants who withdrew from the trial were included in the analysis of outcomes up to the point of withdrawal. Participants were included in the analysis regardless of whether they engaged with the dCBTi intervention.

For the primary outcome, total number of participants and total recruitment period were used to calculate the average recruitment rate per month. Intervention engagement was calculated as the proportion of participants who completed four or more dCBTi sessions. Treatment satisfaction information was grouped into three levels: dissatisfied, neutral, and satisfied.

Continuous outcomes, comprising diary, actigraphy, questionnaire, glucose and other blood biochemical variables, were evaluated for average change from pre‐to‐post treatment. Consistent with the aims of a feasibility trial, we reported 95% CI for change but did not perform formal statistical significance testing. Cohen's *d* effect size was calculated using the within‐subject treatment effect and the standard deviation of change score.

## 4 Results

### 4.1 Feasibility

Ten local primary care practices identified 3474 participants as potentially meeting eligibility criteria and were sent a participant information sheet. Of this number, 242 (7%) completed the online screening and 36 were considered eligible for the study. Nine participants withdrew prior to consent and four did not respond to further contact, leaving 24 participants enrolled. All 24 participants commenced the intervention and completed baseline procedures. One participant withdrew due to changes in personal circumstances and declined to complete post‐assessment outcome measures, resulting in 23 participants (96%) completing the study (see Figure 2 for the Consolidated Standards of Reporting Trials (CONSORT) flow diagram). Study participants included 17 women (70.8%), mean age 65.5 years (SD = 12.4), CES‐D mean of 12.8 (SD = 9.6), ISI score of 15.2 (SD = 4.9), insomnia duration of 15 years (SD = 16.78) and a mean fasting glucose level of 4.9 mmol/L (SD = 1.1) (Table 1).

**FIGURE 2:**
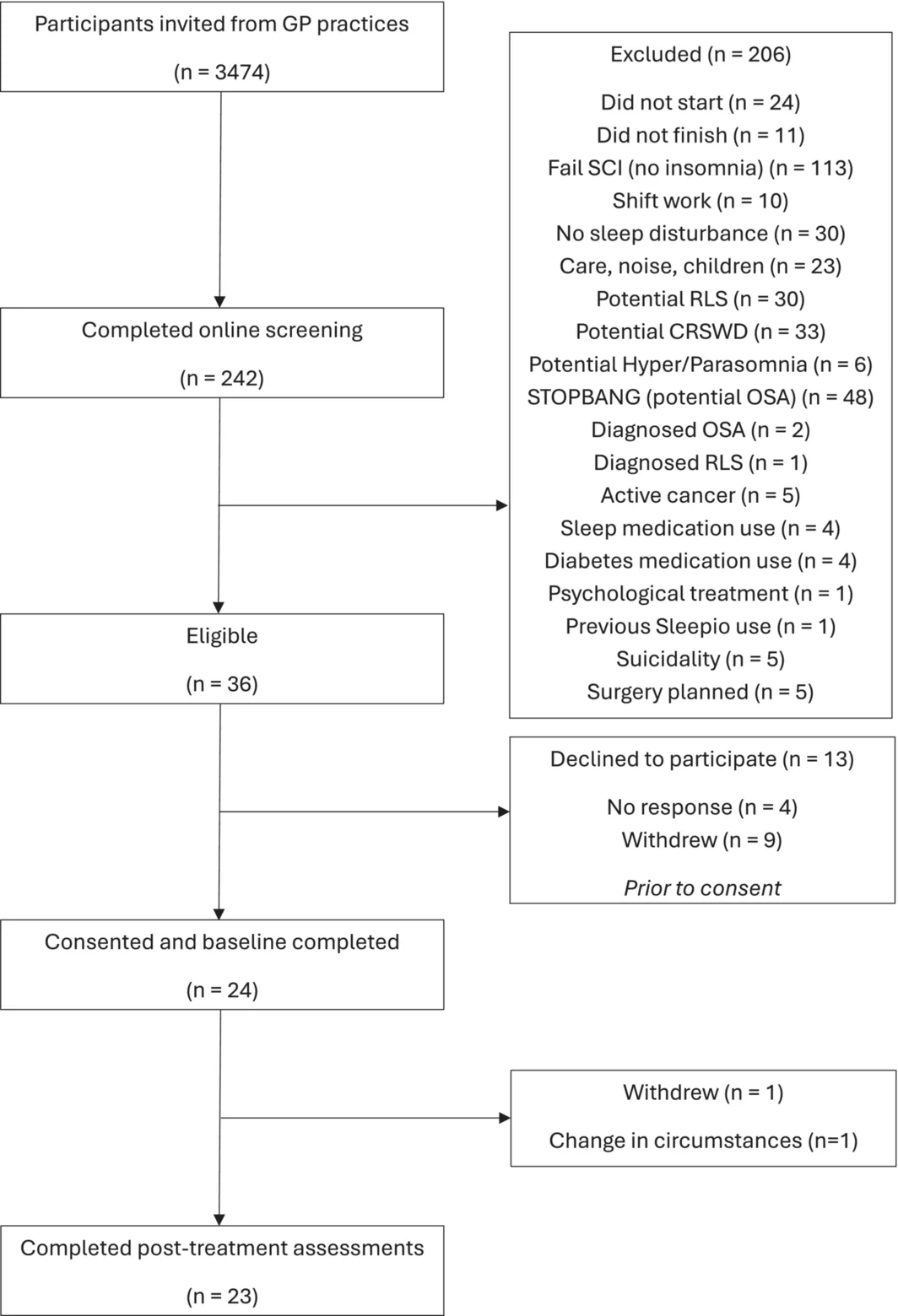
Consolidated Standards of Reporting Trials (CONSORT) flow diagram. Participants could be excluded due to multiple reasons and therefore the number of participants excluded is less than the summation of all the reasons for exclusion.

**TABLE 1:** Baseline characteristics of the participants.

	Overall (*n* = 24)
Gender, *n* (%)	
Male	7 (29.2%)
Female	17 (70.8%)
Age, mean(SD) [min, max]	65.5 (12.4) [30.0 to 88.0]
Marital status, *n* (%)	
Single	3 (12.5%)
Married or in a domestic relationship	19 (79.2%)
Divorced	2 (8.3%)
Education, *n* (%)	
None	2 (8.3%)
GCSE or equivalent	3 (12.5%)
A‐Levels or equivalent	2 (8.3%)
University Undergraduate	2 (8.3%)
University Postgraduate	14 (58.3%)
Prefer not to say	1 (4.2%)
Ethnicity, *n* (%)	
White British	18 (75.0%)
White Other: any other white background	1 (4.2%)
Asian/Asian British: Indian	1 (4.2%)
Asian/Asian British: Chinese	2 (8.3%)
Other Ethnicity, *n* (%)	1 (4.2%)
BMI, mean(SD) [min, max]	25.6 (5.6) [18.9 to 45.7]
Insomnia duration (months), mean(SD) [min, max]	179.7 (201.3) [6.0 to 600.0]

Recruitment took place over 16 months, with an average of 1.5 participants recruited per month. The majority of recruitment occurred in the final 4 months of the study, with an average of 5 participants recruited per month (Figure S1). Slow initial recruitment was due, in part, to a staggered resumption of activities following the COVID‐19 pandemic.

### 4.2 Acceptability

All 24 participants started session 1 of the intervention. Twenty (83.3%) participants completed at least 4 intervention sessions, with two participants completing five sessions and 18 (75%) participants attending all six sessions. Overall, the mean number of sessions attended was 5.3 (SD = 1.5). Seventeen participants (70.8%) were satisfied with the treatment, 6 (25%) were neutral, and no participant reported feeling dissatisfied. Eight participants (33.3%) reported ‘quite a bit’ or ‘a lot’ of improvement in their sleep following treatment. 7 (29.2%) reported ‘somewhat’ of an improvement and 8 (33.3%) reported ‘not at all’ or ‘very little’ improvement.

### 4.3 Proof of Principle Outcome Measures

Improvements in questionnaire‐based outcomes were apparent in some measures from pre‐ to post‐treatment (Table 2), including medium‐to‐large reductions in insomnia symptoms (ISI) [−4.7 (95% CI: −6.2 to −3.2), *d* = −1.4] and depressive symptoms (CES‐D) [−2.7 (95% CI: −5.1 to −0.2), *d* = −0.5]. There was no evidence of change in chronotype (rMEQ) or health‐related quality of life (EQ5D‐3L).

**TABLE 2:** Pre‐intervention and post‐intervention means, standard deviations (SD), pre‐to‐post change scores and effect sizes (Cohen's *d*) for the questionnaire items.

	Pre‐intervention, mean (SD)	Post‐intervention, mean (SD)	Mean change score (95% CI)	Cohen's *d* (95% CI)
ISI	15.2 (4.9)	10.2 (4.8)	−4.7 (−6.2 to −3.2)	−1.4 (−1.8 to −0.9)
MEQr	17.2 (3.3)	17.0 (3.9)	−0.1 (−0.7 to 0.5)	−0.1 (−0.5 to 0.4)
EQ‐5D‐3L index	0.8 (0.2)	0.9 (0.1)	0.0 (−0.0 to 0.1)	0.3 (−0.1 to 0.7)
EQ‐5D‐3L VAS	71.0 (16.6)	74.2 (18.5)	3.6 (−1.3 to 8.6)	0.3 (−0.1 to 0.7)
CES‐D	12.8 (9.6)	9.9 (10.5)	−2.7 (−5.1 to −0.2)	−0.5 (−0.9 to −0.0)

*Note:* 95% confidence intervals (CI) are presented for the mean change and the effect size.

Abbreviations: CES‐D, Centre for Epidemiologic Studies Depression Scale; EQ‐5D‐3L, EuroQol 5 Dimension; ISI, Insomnia Severity Index; MEQr, Morningness‐Eveningness Questionnaire reduced form.

There tended to be greater sleep improvement in diary outcomes compared to the actigraphy data (Tables 3 and 4 and Figure S2). For TIB, the effect was consistent across both actigraphic and diary measures, with a medium reduction of 33.2 min (*d* = −0.5). For total sleep time (TST), actigraphy data revealed a decrease of 18.5 min (*d* = −0.5), whereas the sleep diary data showed an increase of 21.1 min (*d* = 0.5). Self‐reported SOL (*d* = −0.9), SE (*d* = 0.8), sleep quality (*d* = 0.6) and cognitive arousal (*d* = −0.6) improved from pre‐to‐post intervention. There were no other changes in actigraphy‐defined rest‐activity or sleep continuity outcomes.

**TABLE 3:** Pre‐intervention and post‐intervention means, standard deviations (SD), pre‐to‐post change scores and effect sizes (Cohen's *d*) for the actigraphy outcome measures, averaged over the week.

	Pre‐intervention, mean (SD)	Post‐intervention, mean (SD)	Mean change score (95% CI)	Cohen's *d* (95% CI)
TIB	540.5 (64.3)	508.3 (46.3)	−33.2 (−61.9 to −4.4)	−0.5 (−1.0 to −0.1)
TST	444.2 (45.3)	424.9 (43.3)	−18.5 (−36.5 to −0.5)	−0.5 (−0.9 to −0.0)
WASO	72.1 (35.2)	62.4 (25.0)	−8.7 (−22.5 to 5.1)	−0.3 (−0.7 to 0.2)
SE	82.7 (7.6)	83.8 (6.0)	1.3 (−0.5 to 3.2)	0.3 (−0.1 to 0.8)
SOL	9.4 (12.4)	9.6 (8.6)	0.3 (−2.6 to 3.2)	0.0 (−0.4 to 0.5)
RA	0.868 (0.086)	0.885 (0.067)	0.017 (−0.029 to 0.063)	0.3 (−0.2 to 0.7)
IS	0.544 (0.123)	0.535 (0.105)	−0.009 (−0.077 to 0.060)	−0.2 (−0.7 to 0.2)
IV	0.907 (0.210)	0.942 (0.149)	0.035 (−0.074 to 0.144)	0.2 (−0.3 to 0.6)

*Note:* 95% confidence intervals (CI) are presented for the mean change and the effect size. IS: interdaily stability of the rest activity cycle (non‐parametric circadian rhythm analysis).

Abbreviations: IV, intradaily variability of the rest activity cycle (non‐parametric circadian rhythm analysis); RA, relative amplitude of the rest activity cycle (non‐parametric circadian rhythm analysis); SE, sleep efficiency (%); SOL, sleep onset latency (decimal minutes); TIB, time in bed (decimal minutes); TST, total sleep time (decimal minutes); WASO, wake after sleep onset (decimal minutes).

**TABLE 4:** Pre‐intervention and post‐intervention means, standard deviations (SD), pre‐to‐post change scores and effect sizes (Cohen's *d*) for the sleep diary outcome measures, averaged over the week.

	Pre‐intervention, mean (SD)	Post‐intervention, mean (SD)	Mean change score (95% CI)	Cohen's *d* (95% CI)
TIB	540.9 (64.5)	507.7 (46.9)	−33.2 (−61.2 to −5.2)	−0.5 (−0.9 to −0.1)
TST	371.1 (48.9)	392.2 (62.6)	21.1 (1.2 to 41.0)	0.5 (0.0 to 0.9)
WASO	76.3 (42.8)	60.2 (38.2)	−16.2 (−36.2 to 3.8)	−0.3 (−0.8 to 0.1)
SE	69.1 (7.5)	77.5 (10.4)	8.5 (3.9 to 13.0)	0.8 (0.4 to 1.2)
SOL	30.3 (22.7)	18.0 (12.5)	−12.2 (−18.4 to −6.1)	−0.9 (−1.3 to −0.4)
Sleep Quality	1.7 (0.5)	2.2 (0.6)	0.5 (0.1 to 0.8)	0.6 (0.1 to 1.0)
Cognitive arousal	1.7 (0.9)	1.2 (1.0)	−0.5 (−0.8 to −0.1)	−0.6 (−1.1 to −0.2)

*Note:* 95% confidence intervals (CI) are presented for the mean change and the effect size. Sleep Quality: Scored on a 5‐point Likert scale from 0 ‘very poor’ to 4 ‘very good’, with higher average scores indicating better self‐reported sleep quality. Cognitive arousal: Scored on a 5‐point Likert scale from 0 ‘not at all’ to 4 ‘extremely’, with higher scores indicating greater pre‐sleep cognitive arousal.

Abbreviations: SE, sleep efficiency (%); SOL, sleep onset latency (decimal minutes); TIB, time in bed (decimal minutes); TST, total sleep time (decimal minutes); WASO, wake after sleep onset (decimal minutes).

There was a trend towards improvement in most of the fasting blood outcome measures (Table 5 and Figure 3). There was a medium‐to‐large reduction in triglyceride (TAG) levels, as well as medium reductions in lactate (Lact) and glycerol (Glyc) levels. A laboratory processing error resulted in the loss of HbA_1c_ data.

**TABLE 5:** Pre‐intervention and post‐intervention means, standard deviations (SD), pre‐to‐post change scores and effect sizes (Cohen's *d*) for the fasting blood sample outcome measures.

	Pre‐intervention, mean (SD)	Post‐intervention, mean (SD)	Mean change score (95% CI)	Cohen's *d* (95% CI)
TAG (μmol/L)	930.0 (366.3)	780.0 (253.5)	−148.7 (−245.9 to −51.4)	−0.7 (−1.2 to −0.2)
Chol (mmol/L)	4.4 (1.2)	4.2 (1.0)	−0.2 (−0.8 to 0.4)	−0.2 (−0.6 to 0.3)
HDL (mmol/L)	2.6 (1.1)	2.6 (1.2)	−0.0 (−0.5 to 0.5)	−0.0 (−0.5 to 0.5)
OHB (μmol/L)	155.3 (199.0)	146.1 (144.0)	−3.3 (−121.2 to 114.6)	−0.0 (−0.5 to 0.4)
ApoB (g/L)	0.8 (0.2)	0.7 (0.2)	−0.0 (−0.1 to 0.1)	−0.1 (−0.6 to 0.3)
NEFA (μmol/L)	664.2 (416.9)	567.5 (253.3)	−87.4 (−263.6 to 88.8)	−0.2 (−0.7 to 0.2)
CRP (mg/L)	4.2 (2.8)	3.2 (0.5)	−1.0 (−2.2 to 0.2)	−0.4 (−0.8 to 0.1)
Lact (mmol/L)	1.2 (0.5)	0.9 (0.3)	−0.3 (−0.5 to −0.1)	−0.6 (−1.1 to −0.2)
Glyc (μmol/L)	63.0 (35.2)	51.3 (25.6)	−13.1 (−26.7 to 0.5)	−0.4 (−0.9 to 0.0)
Urea (mmol/L)	5.8 (1.8)	6.1 (1.6)	0.5 (−0.1 to 1.1)	0.4 (−0.1 to 0.8)
Glu (mmol/L)	4.9 (1.1)	4.7 (0.7)	−0.2 (−0.7 to 0.3)	−0.2 (−0.6 to 0.3)
Insulin (pmol)	27.9 (23.3)	31.6 (31.4)	−3.3 (−10.1 to 3.5)	−0.2 (−0.7 to 0.2)
ALT (U/L)	46.2 (26.9)	45.0 (17.5)	−3.9 (−14.0 to 6.3)	−0.2 (−0.6 to 0.3)

*Note:* 95% confidence intervals (CI) are presented for the mean change and the effect size.

Abbreviations: ALT, alanine aminotransferase; ApoB, apolipoprotein B; Chol, cholesterol; CRP, C‐reactive protein; Glu, glucose; Glyc, glycerol; HDL, high density lipoprotein; Lact, lactate; NEFA, non‐esterified fatty acids; OHB, β‐hydroxybutyrate; TAG, triglycerides.

**FIGURE 3:**
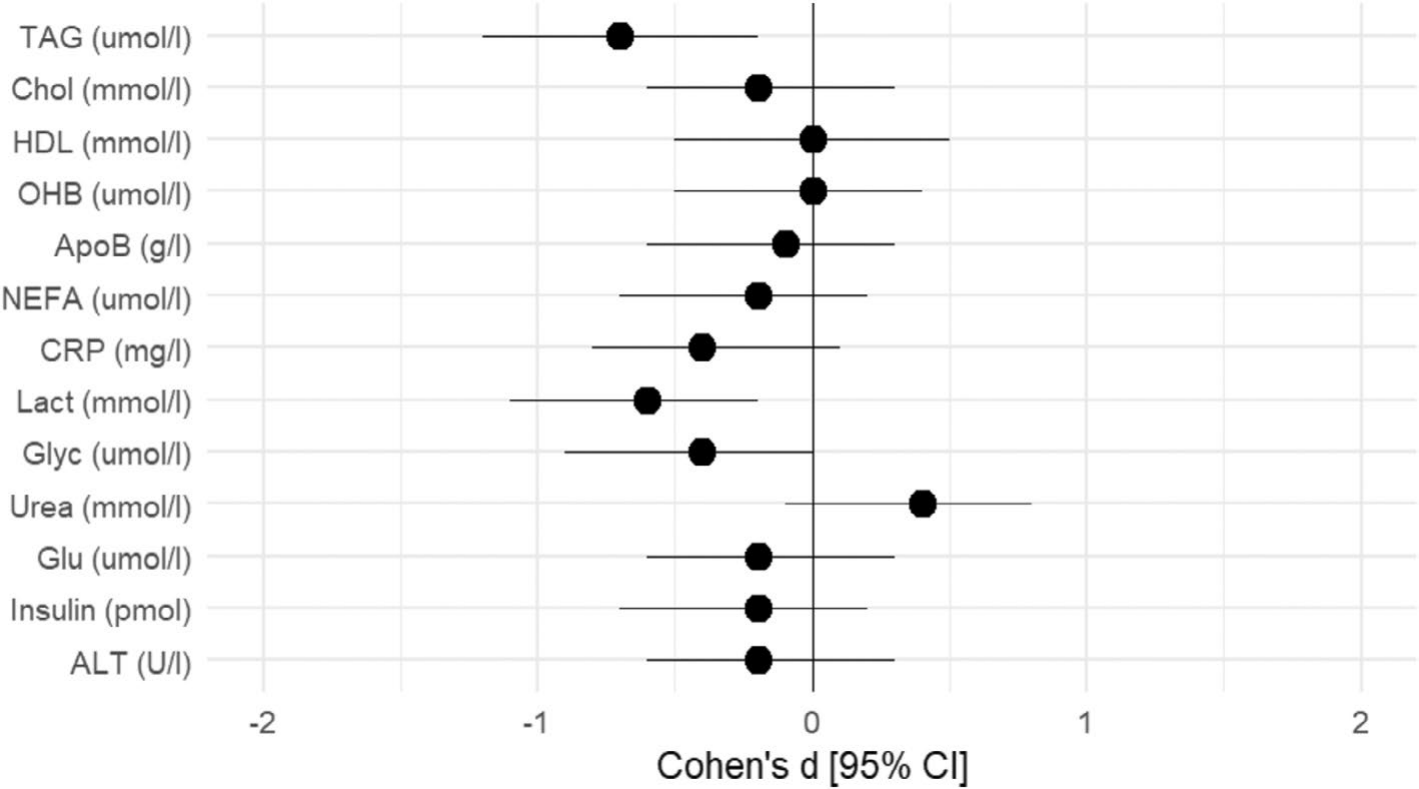
Forest plot showing the 95% confidence intervals of the effect sizes for the fasting blood sample outcome measures. ALT, alanine aminotransferase; ApoB, apolipoprotein B; Chol, cholesterol; CRP, C‐reactive protein; Glu, glucose; Glyc, glycerol; HDL, high density lipoprotein; Lact, lactate; NEFA, non‐esterified fatty acids; OHB, β‐hydroxybutyrate; TAG, triglycerides.

There were no clear pre‐to‐post differences in visuospatial working memory (VSTM) or continuous glucose measures (Tables S1 and S2).

### 4.4 Adverse Events

No serious adverse events were reported to the trial team. There was no evidence of increased rates of pre‐defined adverse events (work‐related accidents, motor vehicle accidents, near‐misses while driving, falling asleep while driving or falls) following the intervention period (Table S3).

## 5 Discussion

This study provides preliminary data to support a future trial of dCBTi in individuals with NDH. Regarding the primary outcome, the recruitment rate was initially slower than expected, which we attribute in part to the COVID‐19 pandemic and challenges in resuming clinical research activities. Initial recruitment saw three participants enrolled between August 2022 and March 2023 from four primary practices. The addition of six practices from August 2023 to November 2023 saw 21 participants enrolled at a rate of 5 participants per month. The addition of six practices between August 2023 and November 2023 led to the enrolment of 21 participants at a rate of 5 per month. There was significant site‐level variation in recruitment and engagement. Three of the first four sites were situated in predominantly university areas and had a smaller number of NDH patients proportionally (1%–1.9%). When recruitment moved beyond these areas to practices with a proportionally higher number of NDH patients (3.1%–5.3% of the total list size), enrolment accelerated, with the majority of participants recruited from three sites (*n* = 16). Another explanation for lower‐than‐expected recruitment might relate to the mode of initial contact. This was done via a posted letter, which included a printed link to the website for the screening questionnaire, requiring participants to manually type the link details into their internet browser. This additional step may have hindered engagement. Future trials should consider tailored recruitment messaging, text and email communications with direct, clickable links and ensuring that recruitment sites are in areas of greatest need. Treatment engagement was excellent (83.3% completed ≥ 4/6 sessions), treatment satisfaction was high (*n* = 17 satisfied, *n* = 6 neutral) and follow‐up rates were high (96%).

Consistent with the broader literature regarding digital sleep interventions, mean change, confidence intervals and effect size estimates indicated positive improvements in symptoms of insomnia and depression (Espie et al. 2012; Henry et al. 2021; Kyle et al. 2020; Tamm et al. 2025). Subjective sleep parameters showed greater improvement than actigraphy‐defined sleep. Although no inferential statistics were conducted, we observed a potential reduction in markers of lipolysis, suggesting improved adipose tissue function and a trend towards reduced systemic inflammation (CRP) following dCBTi. An adequately powered, randomised trial, with refined recruitment strategies, is now indicated to confidently appraise the impact of insomnia treatment on key metabolic outcomes in those at risk of developing T2D.

Several factors should be considered when interpreting our findings. The primary focus of this single‐centre, non‐randomised study was on feasibility and acceptability; therefore, no conclusions can be drawn regarding efficacy. Our recruited sample was highly educated and had a relatively low BMI (25.6) compared with the broader NDH population (e.g., mean of 31.1 within the UK national diabetes prevention programme (Ross et al. 2022)). Our decision to exclude people with a diagnosis of sleep apnoea or those screening positive for OSA (on the STOP‐BANG) likely contributed to sample characteristics. Eligibility screening for this study was conducted entirely via questionnaires and, therefore, may have reduced diversity in the sample due to exclusion through over‐sensitivity and low specificity of the measures. Mean baseline fasting glucose levels turned out to be in the normal range at 4.9 mmol/L (NDH range is 5.6–6.9 mmol/L), potentially limiting scope for improvement post‐intervention. This may relate to our inclusion criteria of having an HbA_1c_ level of 6%–6.4% within the past 12 months or a diagnosis of pre‐diabetes. Future studies may wish to employ higher specificity screening measures, including home‐based assessments of respiratory sleep disorders and conduct a screening measure of blood sugar levels to ensure NDH status prior to study intake. Participation in other lifestyle interventions was not an exclusion; therefore, participants may have been simultaneously engaged in other interventions whilst in this trial, which was not monitored for. Finally, we found a small reduction in actigraphy‐derived TST following the intervention, though a small increase in self‐reported TST. This is likely driven by the sleep restriction component, which uses mild sleep deprivation to support sleep consolidation (Maurer et al. 2020, 2021; Steinmetz et al. 2024). A decrease in actigraphy‐derived TST has been observed in previous CBT studies at post‐treatment (Mitchell et al. 2019), and it may take subsequent extension of the sleep window to deliver improvement in sleep duration at follow‐up. While work is needed to determine what features of insomnia disorder (e.g., regularity, continuity, duration or arousal) are most related to metabolic parameters, investigators should consider assessing both the acute and long‐term outcomes of insomnia treatment in future trials.

Several countries have introduced lifestyle intervention programmes aimed at preventing the conversion from NDH to T2D (for examples see UK (Ross et al. 2022) and USA (The Diabetes Prevention Program (DPP) Research Group 2002)). These programmes primarily focus on improving glycaemic outcomes through caloric control and increasing physical activity. Emerging data identify sleep as a potentially important modifiable factor with causal relevance to metabolic health (Che et al. 2021; Liu et al. 2022; Che et al. 2021; Liu et al. 2022). This feasibility study has demonstrated that a digital sleep intervention for individuals with NDH is both feasible and acceptable. Early signals on sleep, inflammatory and cardiometabolic outcomes now require evaluation in large‐scale randomised controlled trials.

## Author Contributions

**Rachel Sharman:** conceptualisation (equal), data curation (lead), investigation (lead), methodology (equal), project administration (lead), original draft (lead), writing – review and editing (equal). **David Ray:** funding acquisition (equal), conceptualisation (equal), writing – review and editing (equal). **Andrew Farmer:** funding acquisition (equal), conceptualisation (equal), writing – review and editing (equal). **Poppy C. E. Green:** writing – conceptualisation (supporting), visualisation (lead). **Victoria Harris:** formal analysis (lead), writing – review and editing (equal). **Fredrik Karpe:** funding acquisition (equal), conceptualisation (equal), writing – review and editing (equal). **Colin A. Espie:** investigation (support), writing – review and editing (equal). **Diana Mantripp:** investigation (support). **Thomas Marjot:** investigation (support). **Niall M. McGowan:** data curation (support), investigation (support), project administration (support), writing – review and editing (equal). **Jeremy W. Tomlinson:** funding acquisition (equal), conceptualisation (equal), writing – review and editing (equal). **Simon D. Kyle:** funding acquisition (lead), conceptualisation (equal), supervision (lead), writing – review and editing (equal).

## Funding

This study was funded by the National Institute for Health and Care Research (NIHR) Oxford Health Biomedical Research Centre (ref: NIHR203316) and the NIHR Oxford Biomedical Research Centre (ref: BRC‐1215‐20008, Cluster Award 2019–2020). A.F. is supported by the NIHR Oxford BRC. R.S., D.R., P.C.E.G., C.A.E. and S.D.K. are supported by the NIHR Oxford Health BRC.

## Conflicts of Interest

C.A.E. is a co‐founder of, and a shareholder in, Big Health, developer of Sleepio. Big Health Ltd. provided Sleepio at no cost. The CGM devices were provided by Dexcom at no cost. N.M.M. is a current employee of and holds shares, restricted share units and/or share options in Compass Pathways plc; this work is not related to Compass Pathways plc. The other authors declare no conflicts of interest.

## Supporting information


**Table S1:** Pre‐intervention and post‐intervention means, standard deviations (SD), pre‐to‐post change scores and effect sizes (Cohen's *d*) for the visual short‐term working memory task. 95% confidence intervals (CI) are presented for the mean change and the effect size.
**Table S2:** Pre‐intervention and post‐intervention means, standard deviations (SD), pre‐to‐post change scores and effect sizes (Cohen's d) for the continuous glucose monitor. Outcomes were averaged over the sensor days worn. 95% confidence intervals (CI) are presented for the mean change and the effect size. ‘Sleep’ time was defined between the hours of 00:00 and 06:00 whereas ‘wake’ time was defined as 06:01 to 23:59. Glucose levels are recorded in mmol/L.
**Table S3:** Pre‐intervention and post‐intervention *n*, means and standard deviations (SD), pre‐to‐post for the pre‐specified trial adverse events.
**Figure S1:** Bar graph showing enrolment rates per month. The study ran from August 2022 to November 2023.
**Figure S2:** Forest plot showing the 95% confidence intervals of the effect sizes for the matched actigraphic and sleep diary variables. SE, sleep efficiency; SOL, sleep onset latency; TIB, time in bed; TST, total sleep time; WASO, wake after sleep onset.

## Data Availability

The data that support the findings of this study are available from the corresponding author upon reasonable request.
